# 25Hydroxy-vitamin D status in patients with berardinelli-seip syndrome (congenital generalized lipodystrophy)

**DOI:** 10.1186/1758-5996-7-S1-A108

**Published:** 2015-11-11

**Authors:** Lia Beatriz de Azevedo Souza Karbage, Ana Paula Dias Rangel Montenegro, Luciana Felipe Férrer Aragão, Izabella Tamira Galdino Farias Vasconcelos, Virgínia Oliveira Fernandes, Annelise Barreto de Carvalho, Clarisse Mourão Melo Ponte, Catarina Brasil D'Alva, Carla Soraya Costa Maia, Synara Cavalcante Lopes, Marivaldo Loyola Aragão, Carla Antoniana Ferreira de Almeida Vieira, Ana Paula Germano Lopes Cavalcante, Renan Magalhães Montenegro

**Affiliations:** 1 Universidade Federal do Ceará, Fortaleza, Brazil

## Background

Low status of 25-hydroxyvitamin D [25(OH)D] is defined as level< 30ng/mL. Studies indicate high prevalence of hypovitaminosis D at different ages in many regions of Brazil. It is associated with decreased insulin sensitivity and reduced pancreatic β-cell function. Berardinelli-Seip Syndrome (BS) is a rare congenital autosomal recessive disease due to mutations in 4 main genes (AGPAT2, BSCL2, CAV1 and PTRF). It presents lack of metabolically active fat, and ectopic adipose storage in liver and skeletal muscle, impaired metabolism of lipids and carbohydrates, insulin resistance, diabetes mellitus (DM) and dyslipidemia. We did not find in the literature descriptions about levels of 25(OH)D in groups of patients with BS.

## Objective

To describe 25(OH)D blood levels and its correlation with insulin, glycosylated hemoglobin (A1c), HOMA-IR and hepatic steatosis in patients.

## Materials and methods

Cross-sectional study conducted between 2013 and 2014. We evaluated 13 patients with BS followed at a University Hospital. Blood dosage of 25(OH)D, insulin, A1c, HOMA-IR, abdominal ultrasound and genetic study was made. Associations were tested by Spearman's rank correlaction coefficient.

## Results

The results were expressed as median, 25th percentile and 75th percentile for 25(OH)D levels, mean and standard deviation for other laboratory tests, and absolute frequency for the other variables. The median of 25(OH)D was 36.3 (26.3 – 40.5) ng/mL. Nine patients presented normal levels of 25(OH)D, with median of 40.5 (36.3-42.1) ng/mL, 2 presented insufficiency (26 ng/mL) and 2 presented deficiency (18.2 ng/mL). Among other variables, 6 patients had DM, 12 hepatomegaly and 4 hepatic steatosis. All patients had severe low leptin levels (1.2±0.3 ng/mL). Four patients showed mutation on AGPAT2 gene, and 6 on BSCL2. The genetic study of 3 patients is in progress. The correlation coefficients between 25(OH)D levels and insulin, A1c, HOMA-IR, DM, and hepatic steatosis were not significant.

## Conclusion

In this study there was an unexpected predominance of normal levels of 25(OH)D in patients with BS, which is not an usual finding in Brazilian population nowadays. Some authors hypothesize that high leptin levels impairs the synthesis of 25(OH)D in obese. It may suggest a possible role of leptin deficiency in the 25(OH)D sufficiency in BS. Another hypothesis are that excess of subcutaneous fat would sequester vitamin D and that hepatic injury would result in low 25(OH)D synthesis.

